# Schizophrenia risk factors in exceptional achievers: a re-analysis of a 60-year-old database

**DOI:** 10.1038/s41598-018-37484-9

**Published:** 2019-02-04

**Authors:** Andrei Szöke, Baptiste Pignon, Franck Schürhoff

**Affiliations:** 1 AP-HP, Pôle de Psychiatrie des Hôpitaux Universitaires H Mondor, Créteil, F94000 France; 2 INSERM, U955, Equipe 15 Psychiatrie translationnelle, Créteil, F94000 France; 3 Université Paris-Est, Ile-de-France Créteil, F94000 France; 4 Fondation FondaMental, Créteil, F94000 France

## Abstract

Current medical research has focused on diseases and their associated risk factors. As such, these factors are assumed to have a deleterious effect. An alternative hypothesis is that some of these risk factors would also increase the chance for an opposite, positive outcome. To test this hypothesis, we considered exceptional social achievement and schizophrenia as opposite outcomes. Sixty years ago, researchers in France collected data on socio-demographic factors associated with exceptional social achievement. As the number of female subjects in the original database was very limited, we restricted our analyses to men. We tested the odds of achieving prominence in the presence of factors known to be associated with an increased risk of schizophrenia, namely migration, urbanicity, seasonality of birth, birth order, and paternal age. Three of the five factors tested significantly increased the odds for exceptional social achievement (urban birth, being the first-born and father’s age over 35). Our findings suggest that some of the factors that are currently considered as risk factors for schizophrenia could diversifying factors. Widening the focus of research to include all potential effects of factors associated with disease could have important consequences on our understanding of causal mechanisms and for designing public health interventions.

## Introduction

Schizophrenia is one of the most severe forms of mental illness, and is often chronic, recurrent, disabling, and debilitating. Characteristics of schizophrenia typically include positive symptoms, such as hallucinations or delusions, disorganized speech, negative symptoms, such as flat affect or poverty of speech as well as impairment in cognition, including attention, memory and executive deficits. This leads to severe impairments in reality testing, goal-directed activities and social interactions^1^.

Epidemiological and genetic research has led to the identification of several genetic and environmental risk factors for schizophrenia^2^. Genetic risk factors include common variants of small effect as well as rare variants of greater effect^3^. The evidence for some of the environmental factors that have been associated with an increased schizophrenia risk such as migration, urbanicity, seasonality of birth is compelling. Most of them are associated with a moderate increase in relative risk (i.e. in the probability to develop the disease in the exposed group relative to the probability in the unexposed group). However, because they usually affect large proportions of the population, the associated attributable risk (which shows how many extra cases have been caused by this exposure) could be much greater^4^. For some other environmental factors, such as birth order, the relationship to schizophrenia remains controversial, although an increased risk has been reported for first-born subjects^5^. Some of the factors associated with an increased risk of schizophrenia, such as paternal age, are difficult to classify as genetic or environmental. However, it must be noted that the demarcation between genetic and environmental factors is often artificial as both types of factors contribute to the overall risk, often in interaction.

The general underlying assumption behind this research is that genetic and/or environmental factors associated with schizophrenia have a consistent deleterious effect on normal functioning or brain development^6^.

Although this may well be true for some, possibly most, of such schizophrenia risk factors, other risk factors may not be deleterious *per se*. A more encompassing hypothesis would be that some factors are associated with a higher variability of outcomes, i.e. a more frequent occurrence of atypical outcomes, both positive and negative. This could be the case if, for example, these “risk factors” increase the sensitivity of the subject to other (favorable or deleterious) factors, or if they have opposite effects on individuals depending on the specific individual background/characteristics^7,8^.

Such a hypothesis would also offer a potential explanation for the “evolutionary paradox of schizophrenia”^9^. As mentioned earlier, there is an important genetic component to the etiology of schizophrenia. Additionally, the disorder has an early onset, is associated with high social impairment, increased mortality, and reduced reproductive rates^10^. Thus, given all these characteristics, the fact that natural selection has not eliminated the genetic variants that predispose to schizophrenia seems paradoxical. Several explanations have been advanced for the persistence of the disorder and its genetic susceptibility variants in the population but none is completely satisfying^9^. If genetic variants associated with schizophrenia changed the variability of the outcomes but not the mean phenotype, as predicted by our hypothesis, this would help explain, at least in part, why some alleles associated with schizophrenia persist in the population^11^.

The dominant paradigm of risk factors research is the identification of factors associated with disease. Such a paradigm, due to its sole focus on pathology risk and negative outcomes, occludes the potential influence of such factors on positive outcomes, such as may arise from an increase in diversity. A paradigm shift would require the identification of contrasting outcomes. We suggest that exceptional social achievement and schizophrenia could represent such contrasting outcomes. Indeed, achieving prominence usually involves a good grasp of reality, remarkable social and cognitive skills, flexibility and high motivation, all qualities in sharp contrast to the typical clinical presentation in schizophrenia.

The existence of a study on “determinants of (exceptional) social achievement” conducted in France more than 60 years ago, for which the original source data were recently made available, offered us the opportunity to test this hypothesis^12^. The original study analyzed several variables. Some of these variables are of interest for us as they have been associated with an increase in the risk for schizophrenia (e.g. paternal age, migration, urbanicity, birth order).

Some other variables of interest for us, such as seasonality of birth, were not collected and analyzed in the initial study. For some of the variables analyzed, such as migration, matching cases (achievers) and controls (general population) on potential confounders, such as gender and age, required improvement. Finally, for still other data, such as father’s age, improved computing capacity allowed for a more adequate statistical treatment of data. For all these reasons, a reanalysis of the data was deemed necessary. Thus, the aim of this study was to look for associations between factors known to be associated with schizophrenia, namely migration, urbanicity, seasonality of birth, birth order, and paternal age, and exceptional social achievement.

## Methods

### General overview

The present study, a classical case-control study, aims to compare the characteristics of a sample of exceptional achievers (cases) with the characteristics of the general population (controls). The analysis focuses on five demographic characteristics that have been associated with an increased risk for schizophrenia: being first-born, migration, winter-spring birth, paternal age and urbanicity^2,4,5,13–16^. Although for birth rank and migration, definition of the exposure was self-evident, for the other three variables, we had to choose between several possible definitions. Winter-spring births have been associated with an increased risk of schizophrenia^6^. However, the exact period of risk has not been homogeneously defined in all studies, and when monthly risk has been tabulated, results have shown some variability between studies^17^. We thus decided to define our risk period based on the largest study to date^18^, and therefore consider subjects born from December to March as exposed. For father’s age at birth of their child, we based our threshold for defining subjects exposed (i.e. paternal age of 35 or more) on the meta-analysis by Wohl and Gorwood^15^. For urban birth, we used the threshold used to define urban/rural populations in the data sources available (censuses). Thus, all subjects born in towns with a population of 2000 or more have been considered exposed.

Given the available data, samples were as closely matched as possible on potential confounding variables, such as gender, age and year of birth.

### Data

#### Data on achievers (Numerator data)

In 1957, the French National Institute for Demographic Studies (INED) conducted a survey among “prominent people” to study “(exceptional) social achievement in France, its characteristics, laws and consequences”^12^. For this purpose, the researchers sent a short questionnaire by mail, to all the people listed in an official directory of influential/noteworthy people in France (published in 1954) and also to members of parliament and professors from Paris universities^19^.

The questionnaire mainly enquired about demographic characteristics, including birth year and that of their parents, place of birth and birth rank (the complete list of items in Supplementary Table 1). Data for 2070 subjects were thus collected, giving a response rate of 58.3%. The file containing these data was obtained from the “Centre Maurice Halbwachs – ADISP” (www. cmh.ens.fr/greco/adisp.php)^20^.

To protect anonymity of the participants, the exact date of birth was not available in this file. Thus, to study seasonality of birth, we retrieved information on the month of birth of the subjects listed in the aforementioned directory of influential/noteworthy people in France directly from the directory or, when not available (9.3% of the subjects), from the Internet. Thus, we were able to retrieve information also for the subjects that declined to answer the initial, 1957, survey. Using this procedure, we compiled data for 2,889 subjects (which represent more than 99% of the subjects for which information was sought).

Because of this difference in the acquisition of data, the sample used for analyzing seasonality of birth, although largely overlapping with the INED data, is slightly different from the one used for the other analyses. It includes a larger proportion of the subjects listed in the official directory but not the additional subjects contacted in the INED study (members of parliament and professors from the Paris universities) for which the list was not available.

Table 1 summarizes sample sizes, proportion of women and birth year distribution for the samples of achievers used in the analyses (numerator samples).

**Table 1:** Main characteristics of the samples used in the analyses.

		Initial Sample	Seasonality Sample
N		2070	2889
Women (%)		3.80	5.48
Year of birth (%)*	before 1879	5.96	10.01
	1879 to 1888	15.61	17.99
	1889 to 1913	69.38	64.32
	after 1913	9.04	7.65

^*^For men.

As there were several important differences between genders, such as age and occupation, coupled to data on women representing less than 6% of either of the two samples, the analyses were restricted to men.

#### General population (Denominator) data

Data related to birth (seasonality of birth, urbanicity, paternal/maternal age): Data were extracted from official statistical sources for the general population covering the period from 1881 to 1911. We chose this period in order to cover most of the birth years in the samples of achievers (more than two thirds), and to avoid the atypical period of the First World War. Data on number of births per month, parents’ age (combinations of 5-year age bands), and urban births (defined as birth in a town with population greater than 2000) were available.

None of these data were tabulated by gender. However, we are not aware of any data suggesting a link between gender and any of these variables.

We used data from the census years (1881, 1891, 1901 and 1911) and from the middle years of the inter-census intervals (1886, 1896, 1906). Data on parents’ age at their child’s birth were available only from 1892. Thus, for this variable, we used data from the years 1892, 1896, 1901, 1906 and 1911.

Whenever data from the general population showed a secular trend (e.g. proportion of urban births increasing over time), we weighted the general population (denominator) data to match the data from the achievers (numerator) samples.

#### Migration

In order to match data available for achievers, migrant status was defined as born outside France.

The proportion of migrants varies in different age bands and according to gender. To match the structure of the achievers sample, restricted to males, we were interested in the migrant status of men aged above 35 years. To estimate this number, we combined several available sources of information: the 1954 census^21^, other public statistics^22^ and a synthesis of the history of migration in France^23,24^ (details from the authors upon request).

#### Birth rank

Two different theoretical distributions for birth ranks (from 1 to 9 or more) were used as the reference (denominator).

The first was based on data from the general population (the same used by the initial INED study). However, in this case the comparison between achievers and the reference distribution could be biased if differences exist in the two populations on important characteristics that influence family structure (non-comparability). For example, if socio-economic levels in the families of achievers were different from those in the general population and associated with different family structures i.e. number of sibs the role of birth rank would be confounded by the socio-economic level.

Thus, we used for comparison a second sample, based on the distribution of birth ranks in the sample of achievers (restricted to males). Using this distribution allowed us to compare the odds for achievement between siblings from the same families and thus to avoid any potential biases due to familial factors.

As for data related to birth, data from the general population on birth rank were not tabulated by gender. For this reason, and because we were not aware of any data suggesting a link between gender and birth rank, the general population data was not restricted to males.

For the general population distribution, we used data available from the 1931 census, for women born between 1850 and 1889 that had at least one child. More than 90% of the mothers of subjects in the achievers sample were born in this interval. The reasoning behind the choice of the 1931 census data is that by then (almost) all women (born between 1850 and 1889) had reached the final number of their offspring, whilst, at the same time, most were still alive.

For those interested in the general population (denominator) estimations, detailed source data and estimation procedures are available from the authors on request.

### Analyses

For all variables, we calculated the odds ratio (OR) and their 95% confidence intervals (95% CI).

The only adjustment used was for father’s age (adjusted for mother’s age). Given that mother’s age at birth has not been related to an increased risk of schizophrenia, we were not interested in its influence on achievement^25^. On the other hand, maternal and paternal age at birth are usually highly correlated and thus, unless we adjust for the influence of maternal age on achievement, it could bias the estimates of the influence of paternal age. For this reason, we decided, as it has usually been done in studies on paternal age and schizophrenia^25^, to adjust for maternal age when calculating the OR for paternal age.

For analyses of variables related to birth, the data pertaining to the achievers samples were restricted to the subjects that matched the available general population data i.e. born between 1879 and 1913 (for father’s age − 1889 to 1913). We performed all statistical analyses using the software “R”^26^.

## Results

### Data on achievers (numerator)

All data on achievers are, with the exception of month of birth, derived from the original INED sample and are summarized in Table 2. The percentage of birth by month, in exceptional achievers, is summarized in Table 3.

**Table 2:** Data (N) on exceptional achievers (restricted to men).

Variable	Exposed	Total	% exposed
Urban birth	1303	1546	84.28
Fathers’ Age >35	473	1330	35.56
Migration*	145	1742	7.68
First-born	295	1972	48.15

^*^Subjects over 35 years old.

**Table 3:** Seasonality (month) of birth in the general population and the achievers’ sample.

% total births by month	1	2	3	4	5	6	7	8	9	10	11	12	% exposed (December to March)
Achievers*	8.91	7.23	8.87	8.51	9.05	8.03	8.56	8.34	8.2	9.71	7.18	7.41	32.42
General Population	8.67	8.33	9.17	8.75	8.58	8.08	8.33	8.25	8	7.92	7.75	8	34.17

*Data for a total of 2255 subjects.

### General population data (denominator)

#### Seasonality of birth

The estimated percentage of births by month for the period 1879 to 1913 is shown in Table 3 (for both achievers – numerator –, and the general population – denominator).

#### Urbanicity

Data shows a steady increase in the proportion of urban births over the period of interest (1879 to 1913). Consequently, we weighted the data (denominator) to reflect the distribution of age/year of birth in the INED sample. The percentage of subjects born in urban areas was thus estimated at 59.35%.

#### Parents’ age at birth of their child

There is a slight tendency towards more births to younger parents for the period of interest (1889 to 1913). Therefore, we weighted the general population data to reflect the distribution of years of birth in the sample of achievers.

The estimated percentage of births by father’s age (by 5 year bands) are presented in Table 4.

**Table 4:** Father’s age at birth (%).

Fathers’ age	<25	25–29	30–34	35–39	40–44	45–49	>50	Exposed (>35)
Achievers	5.1	26.4	32.9	20.0	9.6	3.3	2.6	35.6
General population*	6,3	28.6	27.7	19.3	11.1	4.9	2.1	37.5

*Between1892 and 1913.

#### Migration

The number of men over 35 years, born outside mainland France and living in France in 1954 was estimated at 751,014, which represents 8.1% of the men over 35 living in France at that moment.

#### Birth rank

The percentages of children according to birth rank in the sample of achievers, and in the two different comparison groups (general population and the families of the achievers) are shown in Table 5. Data in Table 5 suggest that, possibly in relation with other characteristics (urbanicity, socio-economic level, etc.), the number of sibs in the families of achievers was lower than in the general population.

**Table 5:** Birth rank (%).

Birth Rank	Achievers	Achievers’ Families	General Population
1	48.15	43.51	30.37
2	25.69	28.55	23.58
3	14.1	14.63	16.09
4	6.15	6.89	10.68
5	3.28	3.2	7.07
6	1.18	1.65	4.74
7	0.77	0.81	3.16
8	0.26	0.43	2.10
≥ 9	0.41	0.34	2.22

### Comparisons between achievers and the general population

Table 6 shows the percentage of achievers and general population subjects exposed to each of the risk factors as well as the OR.

**Table 6:** Comparison between achievers and reference population.

Variable	Achievers (percentage exposed)	General population (percentage exposed)	OR (95% CI)	p
Seasonality of birth (December to March)	32.56	34.15	0.98 (0.90–1.06)	0.14
Urban birth^a,b^	84.28	59.35	7.86 (6.86–9.02)	<0.0001
Father’s Age >35^a,b^	35.56	37.52	1.19 (1.05–1.34)^c^	0.006
Migration	7.68	8.10	0.94 (0.80–1.12)	0.52
First-born	48.15	30.37	2.13 (1.95–2.33)	<0.0001
		43.51	1.20 (1.06–1.36)^d^	0.004

^a^Achievers sample restricted to the subjects that match the general population data (see text for details); ^b^general population data weighted to a similar pattern as the sample of achievers;

^c^after adjustment for mother’s age;

^d^in the families of achievers, limited to men.

#### Seasonality of birth

The OR shows that there is no difference in the proportion of exposed subjects (i.e. subjects born between December and March) between the two samples (OR = 0.98, 95% CI 0.90–1.06), i.e. seasonality of birth was not associated with achievement.

#### Urbanicity

The OR shows that urbanicity at birth significantly increases the odds to achieve social prominence (OR = 7.86, 95% CI 6.86–9.02).

#### Father’s age at birth of their child

The OR for fathers older than 35 years, after adjustment for mother’s age (binary variable using the same 35 years threshold) suggests a slight, but significant, association between achievement and older father’s age (OR = 1.19, 95% CI 1.05–1.34).

#### Migration

The OR suggests that no difference exists, for this variable, between achievers and the general population (OR = 0.94, 95% CI 0.80–1.12).

#### Birth rank

Both comparisons (with data from the general population and from their own families) suggest that being first-born is associated with better odds of achieving prominence (OR = 2.13, 95% CI 1.95–2.33 and OR = 1.20, 95% CI 1.06–1.36, respectively).

## Discussion

In the present study, we used a 60-year-old database to explore an innovative hypothesis: the idea that the same factor could be associated to opposite/contrasted outcomes. To test this hypothesis, we explored the association of socio-demographic factors linked to the risk of schizophrenia with exceptional social achievement. Of the five factors studied, two (seasonality of birth and migration) did not show a significant association. The other three factors (urban birth, advanced paternal age and being first-born) showed an association with exceptional social achievement in the same direction as with schizophrenia. These findings suggest that these factors are not deleterious *per se*, but enhance variability and the probability of extreme (positive or negative) outcomes.

It is important however to note that the present analyses have limitations inherent to the general design of the study and the fact that it has been carried out long after the data was collected.

Case-control (or case-referent) studies are retrospective, and thus depend on the quality of data recording. They could also be subject to bias and non-comparability (between cases and controls)^27^. On the other hand, they are more time and effort efficient and in the case of very rare outcomes (as in the present study), the sole viable option^28^. To minimize the risk of bias, we took several steps to ensure comparability between achievers and the general population for all potential confounders (e.g. age, gender, year of birth) as best as possible, given the available data.

Another potential limitation of the present study is that the factors associated with some of the variables explored (e.g. urbanicity, migration) could have changed over time and most data linking these factors to schizophrenia are more recent. As an example, air pollution associated with urban living was different 60 years ago. However, for all the factors studied here, the first published associations with schizophrenia predate the publication of the original study by the INED. Furthermore, with the exception of paternal age^29^, in the first half of the 20^th^ century, the findings were already confirmed by several studies (see references in Stompe *et al*. 1999^30–33^). Despite this remarkable stability of the findings, a more recent investigation of the association between achievement and these (and other) factors is needed to overcome this potential limitation of our study.

Any generalization of these findings has also to take into account that the sample analyzed was restricted to men and that not all eligible subjects were included (i.e. answered the survey).

The initial 1957 study provided comparisons between achievers and the general population for four of the five variables that we analyzed: migration, urbanicity, father’s age and being first-born. There are however several important methodological differences between our study and the initial (INED) study which justify the reanalysis of the data. Firstly, in the 1957 study, only basic descriptive statistics (mean, percentages) were reported and differences were not tested for statistical significance. Secondly, for some of the analyses, the two populations were not matched (e.g. for migration: the control sample was not restricted to subjects over 35 years) or comparison was not adjusted for important confounders (i.e. for mother’s age when analyzing the role of paternal age). Nevertheless, with the exception of migration (for which the initial study reported a higher percentage of migrants among the achievers), the conclusions from that study are concordant with the present investigation.

In our study, urbanicity showed a very large OR for achievement, and this deserves a more detailed discussion. Urban environment is complex and urban birth should be viewed rather as a marker of increased risk than a risk factor *per se*. Urban environment is associated with increased population density, pollution, noise, increased infectious risk, more social stress, less physical activity, but also more social interactions, and better access to health care and education^34^. Although the specific factors associated with urbanicity that increase the risk for schizophrenia are not known, several lines of evidence point to a very early influence (around the time of birth)^35^. Similarly, our data relate to the place of birth. Although the direction of the effect is, as we hypothesized, in the same direction as for schizophrenia, it is possible that the association is due to different factors (e.g. increased infectious risk for schizophrenia and better access to education for achievement). Unless the exact factors associated with each outcome are uncovered, this possibility could neither be confirmed nor ruled out.

One particular decision that deserves discussion is the choice of the threshold to define urbanicity. The threshold used in this study (2000 people) was imposed by the availability of data and it would seem rather small. However, it is of note that, at the time, almost half of the population was born in rural areas when this definition was used (48,9% in 1901). Furthermore, if an urbanicity factor were to be present only in larger cities (for example with more than 10,000 people), the OR we calculated would actually be an underestimate of the true effect.

Of the demographic factors that are of interest for the present study, the only one already known as related to exceptional achievement in the literature is birth rank. The literature on this topic is abundant, suggesting that first-born subjects are more often found among exceptional achievers. Earlier studies have been criticized for their methods (selection of the achievers group, matching denominator group and statistical methods)^36^; but more recent and rigorous studies, as well as recent reviews of the literature, have confirmed this effect^37,38^. However, on the other hand, it must be stressed that being the first born is, among the factors studied here, the factor for which the evidence of an association with the risk for schizophrenia is the weakest. Not all studies found an increase in risk associated with birth rank. However, when they did it was, with a very few exceptions, associated with being the first-born (see Stompe *et al*.^30^ for a review of studies). In several of these previous studies the excess of risk in first-borns was limited to men. Consistent with this data, the largest study to date (at our knowledge) – based on the study of the Northern Finland 1966 birth cohort – also found an excess of risk for schizophrenia in first-born males^5^.

It is also of interest that two other potential risk factors for schizophrenia (not studied here) have been related to exceptional achievement and may be diversifying factors: childhood traumatic experiences and family history of psychosis^39–42^.

Three of the factors tested in our study (urban birth, being first-born, and having a father older than 35 years), showed an association in the predicted direction, i.e. the same direction for both positive and adverse outcomes. The discussion below focuses on these findings and their potential explanations.

First of all, several decades ago, Meehl convincingly argued (and Standing *et al*.^43^ empirically illustrated) that in psychology, the social sciences, and biology “everything correlates to some extent with everything else” when subject characteristics are involved^44^. Thus, it could be argued that our findings are trivial and a consequence of what Meehl had called the “crud factor”. However, our approach was different from the null hypothesis testing that Meehl criticized. We opposed two alternative hypotheses. The first is the “classical” hypothesis of uniformly deleterious effects of the risk factors, and the second is the hypothesis of diversifying factors that predicted same direction effects on the contrasted outcomes. Our conclusions are thus based mainly on the direction of the differences rather than the statistical significance of the tests. Furthermore, if one accepts our premise of schizophrenia and social achievement as being at opposite ends of a continuum of outcomes, a monotone correlation (as predicted by Meehl’s crud factor) would be consistent with the first (“classical” i.e. risk factor) hypothesis contrary to what we observed.

A second possible explanation is one of a spurious similarity in the associations with the two outcomes. Indeed, the studied variables could be seen more as markers of increased risk, associated with numerous other factors. One of those factors could be the effective factor for an outcome, and a different one could be the effective factor for the contrasted outcome (as suggested above in the discussion on urbanicity). Thus, the two outcomes would be both related to the same variable (marker) but for different reasons.

Another interpretation could be that, even if the effective factor is the same for the two outcomes, its effects are different, and perhaps even opposed, depending on the individual’s characteristics. For example, being the first-born could lead to more demands and responsibilities. The impact of this on factors, such as self-esteem, emotional mastery and autonomy, and finally on achievement will obviously depend on the capacities of the individual to respond to/master the demands.

However, these hypotheses (of spurious similarity or different individual characteristics) imply, for each variable associated with opposing outcomes, the existence of specific, particular explanations/mechanisms. The multiplicity of instances in which opposing outcomes are associated with the same factor would suggest, according to the Occamian simplicity principle, that a common mechanism could be a better explanation.

There are several theoretical frameworks, that could be seen as variations of the same idea, that could explain these findings. In the field of research on exceptional achievement, Simonton called such external factors “diversifying experiences”^45^. Feinberg and Irizarry suggested that genetic factors could also influence the propensity to phenotypic variability, without changing the mean phenotype in a population^11^. The biological sensitivity to context and the differential susceptibility to context theories both suggest that, based on genetic background and environmental factors, some individuals are more susceptible than others to both negative and positive influences (and thus outcomes)^7^. In light of these theories, the factors that we found associated with exceptional achievement could be “diversifying factors” that promote higher susceptibility to the effect of both positive and negative influences. The net result would be more diverse outcomes and a higher proportion of extreme outcomes.

Some authors have already suggested that genetic risk factors for psychosis would also predict high achievement and/or creativity^40,46^. This fact could explain the “evolutionary paradox of schizophrenia”, and why alleles conferring risk for disorders reducing fertility, such as schizophrenia, may persist through balancing selection, as their negative effects are offset by potential benefits^9,47,48^.

The mechanisms through which environmental diversifying factors exert their action are, at this point, speculative but epigenetic mechanisms are obvious candidates^11,49^. It is of interest that childhood trauma, a factor that has been associated both with exceptional achievement and schizophrenia, has also been linked to epigenetic changes^50^. Epigenetic changes in genes related to dopaminergic functioning, which have been linked both to schizophrenia and (scholastic) achievement, could explain our findings^47,51^.

Although this hypothesis seems attractive, a number of points should be noted. Firstly, our findings have to be confirmed and extended to female subjects and other risk factors. Studies allowing for a large and adequate selection of risk and confounding factors to be measured are necessary. As more of these factors are identified, studying their commonalities and interactions could indicate the mechanisms by which they exert their actions.

In addition, epigenetic studies (such as DNA methylation and histone modifications) and gene-environment interaction studies are likely to illuminate the biological underpinnings driving the association of high achievement with environmental factors that increase the risk of schizophrenia.

The studies using this framework of related hypotheses (diversifying experiences, differential susceptibility, sensitivity to context) have mainly dealt with psychological/behavioral outcomes. Although nothing seems to oppose the existence of the same mechanism in the development and pathology of other organs/systems, this remains to be investigated. As indicated in the introduction, one challenge is to define what is an exceptionally positive outcome.

A related question pertains to the specificity of the effect of diversifying factors. Although direct evidence is currently lacking, studies using the “risk factor - negative outcome” paradigm suggest that, at least for some factors, there is no domain specificity (e.g. studies of the Dutch famine found an increased risk for cardio-vascular diseases, obesity, diabetes and schizophrenia^52–55^).

In conclusion, we found that several factors (or markers) associated with an increased risk for an unwanted outcome (schizophrenia) are also associated with a positive outcome (exceptional social achievement). This finding points to the need to assess all possible consequences, positive or negative associated with a given factor. If confirmed, this could have several important consequences: for research, our understanding of mechanisms of normal and pathological development and for the way preventive strategies are designed and their results assessed.

## Supplementary information

Supplementary Table 1. The list of data collected in the initial (1957) study
