# Supplementary material for: Schizophrenia risk factors in exceptional achievers: a re-analysis of a 60-year-old database
Source: Sci Rep. 2019 Feb 4;9:1294. doi: 10.1038/s41598-018-37484-9 (PMC6362112; doi:10.1038/s41598-018-37484-9)
Supplement: Supplementary file 1 — Supplementary Table 1. The list of data collected in the initial (1957) study [file 41598_2018_37484_MOESM1_ESM.pdf]

Schizophrenia risk factors in exceptional achievers: a re-analysis of a 60-year-old database

Andrei Szoke[1,2,3,4], Baptiste Pignon[1,2,3,4], Franck Schürhoff [1,2,3,4,*]

[1] AP-HP, Pôle de Psychiatrie des Hôpitaux Universitaires H Mondor, Créteil F94000, France ;
[2] INSERM, U955, Equipe 15 Psychiatrie translationnelle, Créteil F94000, France ;
[3] Université Paris-Est, Créteil F94000, France ;
[4] Fondation FondaMental, Créteil F94000, France.

*Corresponding author:
Hôpital Albert Chenevier, Groupe hospitaliers Henri-Mondor, CHU de Créteil, Assistance Publique-Hôpitaux de Paris (AP-HP), 40 rue de Mesly, 94 000, Créteil, France
franck.schurhoff@inserm.fr
☎: + 33 1 49 81 31 31; Fax: +33 1 49 81 30 59

**Supplementary Table 1. The list of data collected in the initial (1957) study**
1. Concerning the subject<br>Name/ First name[a]<br>Date of birth[b]<br>Number of siblings<br>Birth rank<br>Place of birth<br>Number of children<br>Education (most advanced degree and age at quitting school)<br>Profession(s)<br>Other abilities (special talents, hobbies) [a]
2. Concerning the parents (separately for the father and the mother)<br>Year of birth<br>Place of birth<br>Origin<br>Number of siblings<br>Profession (last)
3. Special abilities in sibs and ancestors[a]
4. Essential factor for achievement (subjective assessment) [a]

Legends: [a] data not made available; [b] only year of birth made available.
